# Bioactive Composition and In Vitro Antioxidant Properties of Wild Blackberry (*Rubus* spp.) and Blueberry (*Vaccinium* spp.) Populations

**DOI:** 10.3390/molecules31213811

**Published:** 2026-10-22

**Authors:** Fatma Yaylacı Karahalil

**Affiliations:** 1 Department of Chemistry and Chemical Processing Technologies, Biochemistry Program, Maçka Vocational School, Karadeniz Technical University, Maçka, 61750 Trabzon, Türkiye; fyaylaci@ktu.edu.tr; 2 Bee and Bee Products Application and Research Center (Apimer), Karadeniz Technical University, Ortahisar, 61080 Trabzon, Türkiye

**Keywords:** Blackberry (*Rubus* spp.), Blueberry (*Vaccinium* spp.), DPPH, FRAP, phenolic, RP-HPLC-PDA

## Abstract

This study comparatively evaluated the phenolic profiles and antioxidant capacities of wild blackberry (*Rubus* spp., n = 5) and blueberry (*Vaccinium* spp., n = 4) fruits naturally grown in the high-altitude Akılbaba Region (Giresun) of the Eastern Black Sea region (Türkiye). Methanolic extracts of fresh fruits were assessed for total phenolic content (TPC), total flavonoid content (TFC), and antioxidant activities using, the ferric reducing antioxidant power (FRAP) and DPPH assays, while individual phenolic compositions were quantified against 25 standards using RP-HPLC-PDA. The mean TPC and TFC values were determined as 4.49 ± 0.29 mg GAE/g FW and 0.06 ± 0.03 mg QE/g FW for blackberry, and 5.86 ± 1.25 mg GAE/g FW and 0.47 ± 0.13 mg QE/g FW for blueberry, respectively. Regarding antioxidant activity, FRAP values were determined as 96.08 ± 9.92 µmol FeSO_4_∙7H_2_O E/g FW for blackberry and 95.30 ± 13.26 µmol FeSO_4_∙7H_2_O E/g FW for blueberry samples. In the chromatographic analysis, blackberry was primarily distinguished by substantial concentrations of ellagic and gallic acids, while blueberry was characterized by chlorogenic acid as the major phenolic constituent. In contrast, chrysin and pinocembrin were detected as common flavonoid markers in all samples. Overall, although these two wild berry groups exhibited marked differences in their individual phenolic profiles, their antioxidant responses were assay-dependent, with comparable FRAP values but significantly stronger DPPH radical-scavenging activity in blackberry.

## 1. Introduction

In their natural habitats, wild berry species are perennial, woody or semi-woody shrubs that are generally adapted to cool and humid climates. *Rubus* spp. and *Vaccinium* spp., including blackberries and blueberries, are commonly found in acidic, organic matter-rich soils, especially in high-altitude and undergrowth ecosystems [1,2]. While these genera exhibit differences in leaf, flower, and fruit morphology, they play important roles in the sustainability of natural ecosystems through their pollination biology and seed-dispersal mechanisms. Blackberry and blueberry varieties with purplish to dark-colored fruits have long attracted scientific and nutritional interest due to their unique taste and aromatic properties, as well as the potential health benefits they offer thanks to the biologically active compounds they contain [3,4,5].

Blackberries and blueberries are prominent small fruits with extensive applications across the food, pharmaceutical, biomedical, and cosmetic industries, owing to their rich phytochemical profiles and potent antioxidant capacities [4,5]. Beyond fresh consumption, they serve as crucial sources of bioactive compounds in processed products such as juices, purées, jams, fermented beverages, and dried formulations.

While their high anthocyanin content presents a viable natural alternative to synthetic colorants and additives [6], their abundance of polyphenols, flavonoids, and dietary fibers supports their widespread incorporation into nutraceutical products and dietary supplements in the form of standardized extracts [7].

They are widely recognized for their antioxidant, anti-inflammatory, antimicrobial, and anticancer properties, which are largely attributed to their rich content of bioactive compounds [8,9]. These fruits are particularly abundant in polyphenols including anthocyanins, flavonoids, ellagic acid, and tannins, which contribute to their pharmacological potential. Among these, anthocyanins, responsible for the characteristic dark coloration of the fruits, play a central role in free radical scavenging and the mitigation of oxidative stress associated with chronic diseases. In addition, other phenolic constituents such as ellagic acid and flavonoids further enhance the antioxidant and anti-inflammatory activities of *Rubus* spp. and *Vaccinium* spp., through multiple biochemical mechanisms. underscoring their significance as functional foods with health-promoting properties [4,10,11,12,13]. In addition to their nutritional value, both species exhibit distinct pharmacological activities. Dietary intake of blueberries has been documented to improve cognitive performance and attenuate neurodegenerative decline in elderly populations. Concurrently, blackberry extracts exert significant anti-inflammatory and cytoprotective effects demonstrating substantial therapeutic efficacy against chronic inflammatory and oxidative stress-related pathologies [14,15].

Interest in dark-colored small fruits has increased markedly worldwide; however, despite growing attention to *Rubus* spp. and *Vaccinium* spp. in phytotherapy and natural product research. comprehensive studies linking their phytochemical profiles to high-altitude habitats remain limited. This study aimed to comparatively characterize the phenolic composition, total phenolic and flavonoid contents, and in vitro antioxidant properties of wild *Rubus* spp. and *Vaccinium* spp. accessions collected from the Akılbaba Region in the Eastern Black Sea region of Türkiye, while exploring their potential associations with the geographical and topographic characteristics of the sampling site.

## 2. Results and Discussions

Detailed information on the nine forest fruit samples analyzed in this study is summarized in Table 1. All samples were taxonomically identified by forest botanists as belonging to the genera *Vaccinium* spp. and *Rubus* spp. The table includes precise geographic coordinates and collection altitudes ranging from 1588 to 1831 m. corresponding to sampling sites located in the Akılbaba Region in the Eastern Black Sea region of Türkiye.

Table 2 presents the total phenolic content and antioxidant activity of the methanolic extracts. In this study, 70% methanol was used instead of aqueous extracts primarily because aqueous solutions may present technical difficulties during evaporation and are more susceptible to microbial spoilage. Although 70% methanol is less polar than pure water, it is one of the organic solvents with polarity closest to that of water and is therefore widely considered an appropriate and effective solvent for the extraction of phenolic compounds [16]. Indeed, previous studies on berry fruits provide strong support for this hypothesis [17,18].

The mean total phenolic content of the five *Rubus* spp. accessions was 4.49 ± 0.29 mg GAE/g, with accession-level means ranging from 4.23 to 4.80 mg GAE/g. In comparison, the four *Vaccinium* spp. accessions exhibited a higher mean TPC of 5.86 ± 1.25 mg GAE/g, with accession-level means ranging from 4.35 to 7.35 mg GAE/g. However, the difference in TPC between the two berry groups was not statistically significant (*p* = 0.1111).

When compared with the findings from the recent literature on other *Vaccinium* spp., these values fall within the range reported for several other berry species. For instance, studies on *V. macrocarpon* (cranberry) and *V. corymbosum* (blueberry) have shown a similar variability in phenolic content with typical values ranging from 4 to 8 mg GAE/g [19,20]. When comparing the average TPC values of the two berry groups, *Vaccinium* spp. exhibited a higher mean TPC than *Rubus* spp.; however, this difference was not statistically significant (*p* = 0.1111). These variations may be associated with differences in growing conditions, geographical origin, maturity, and other environmental factors. Indeed, various studies in the literature have shown that the secondary metabolite profiles of berry species can vary considerably depending on climatic conditions, geographical origin, and environmental factors [21,22,23,24].

The total flavonoid content of the blackberry samples ranged from 0.02 to 0.11 mg QE/g, whereas the blueberry samples exhibited substantially higher concentrations, varying between 0.34 and 0.58 mg QE/g. The mean TFC was 0.06 ± 0.03 mg QE/g for *Rubus* spp. and 0.47 ± 0.13 mg QE/g for *Vaccinium* spp., and the difference between the two groups was statistically significant (*p* = 0.0159). These findings clearly demonstrate that blueberry samples possess a significantly higher flavonoid content than blackberry samples. Flavonoids represent a major subclass of polyphenols and typically exhibit broader and more potent biological activities compared to simple phenolic acids. These metabolites are well-recognized for their strong antioxidant, anti-inflammatory, and cytoprotective properties [25].

The antioxidant capacities of the samples were evaluated using two different methods. One of these methods, the FRAP (Ferric Reducing Antioxidant Power) method, is based on the reduction of the Fe (III)–TPTZ complex to the Fe (II)–TPTZ complex. The FRAP method is considered a simple, fast, and reliable analytical technique widely used in determining the total antioxidant capacity of natural products [26]. High FRAP values indicate that the samples have a high antioxidant capacity and greater reducing capacity. In the present study, the mean FRAP values were 96.08 ± 9.92 µmol FeSO_4_∙7H_2_O E/g FW for *Rubus* spp. and 95.30 ± 13.26 µmol FeSO_4_·7H_2_O/g FW for *Vaccinium* spp. The difference between the two groups was not statistically significant (*p* = 1.0000).

The TPC values reported in the literature [27] ranged from 1.73 to 3.79 mg g^−1^ for blackberries and 0.77–5.42 mg g^−1^ for blueberries, whereas our samples showed 4.49 ± 0.29 and 5.86 ± 1.25 mg g^−1^, respectively. Similarly, the reported FRAP values ranged from 35.05 to 70.41 μmol g^−1^ for blackberries and 7.41–57.92 μmol g^−1^ for blueberries, while our values were considerably higher at 96.08 ± 9.92 and 95.30 ± 13.26 μmol FeSO_4_·7H_2_O g^−1^ FW, respectively. These differences may be related to variations in cultivar, growing conditions, maturity, and extraction procedures.

The total phenolic content (TPC) of our blueberry sample was 5.86 ± 1.25 mg GAE/g, which was considerably lower than the value of 26.78 mg GAE/g reported by Barut et al. (2019) [21] for *Vaccinium arctostaphylos*. Similarly, the total flavonoid content was 0.47 ± 0.13 mg QE/g in our study blueberry sample, compared with 7.80 ± 0.44 mg QE/g reported by Barut et al. [21]. DPPH free radical scavenging is an analytical technique based on the elimination of free radicals present in the environment and, unlike the FRAP method, low SC_50_ values indicate high antioxidant capacity [28]. In this study, the average SC_50_ values were determined as 0.49 ± 0.04 mg/mL in the blackberry *Rubus* spp. and 2.68 ± 0.09 mg/mL in the blueberry *Vaccinium* spp. The findings reveal that the *Rubus* spp. extracts exhibited significantly lower SC_50_ values than the *Vaccinium* spp. extracts (*p* = 0.0159), indicating stronger DPPH radical-scavenging activity. The higher SC_50_ value observed for our *Vaccinium* spp. samples compared with the value reported by Barut et al. [21] (0.211 ± 0.011 mg/mL) indicates lower radical-scavenging activity. Such variations may be attributed to differences in genotype, geographical origin, extraction solvent, and whether the results are expressed on a fresh- or dry-weight basis.

A notable difference was observed between the responses obtained from the two antioxidant assays employed in this study. While the FRAP values did not differ significantly between *Rubus* spp. and *Vaccinium* spp. (*p* = 1.0000), the DPPH radical-scavenging assay showed a significant difference between the two groups (*p* = 0.0159). The lower SC_50_ values observed for Rubus spp. indicate stronger DPPH radical-scavenging activity compared with *Vaccinium* spp. Such differences may be related to the distinct mechanistic principles of the two assays and the compositional complexity of the extracts. The FRAP assay primarily reflects reducing capacity based on electron-donating ability, whereas the DPPH assay evaluates the ability of compounds to scavenge a stable free radical. Therefore, differences in the qualitative composition and chemical characteristics of phenolic constituents may contribute to the different antioxidant responses observed in the present study [27,29]. In addition to phenolic compounds, other non-phenolic antioxidant constituents may also contribute to the antioxidant capacity of plant extracts [18]. However, these compounds were not determined in the present study, and their contribution cannot be directly evaluated.

Interestingly, the re-evaluated statistical analysis provided a more precise interpretation of the differences between the two wild berry groups. Although *Vaccinium* spp. exhibited a higher mean TPC than *Rubus* spp., this difference was not statistically significant (*p* = 0.1111). In contrast, the TFC of *Vaccinium* spp. was significantly higher than that of *Rubus* spp. (*p* = 0.0159). Despite this difference in TFC, the FRAP values remained comparable between the two groups, with no statistically significant difference (*p* = 1.0000). Conversely, Rubus spp. exhibited significantly lower DPPH SC_50_ values than *Vaccinium* spp. (*p* = 0.0159), indicating stronger DPPH radical-scavenging activity. These findings suggest that antioxidant activity is not determined solely by the total amount of phenolic or flavonoid compounds. The higher TFC of *Vaccinium* spp., together with its comparable FRAP activity but weaker DPPH radical-scavenging activity, suggests that the qualitative composition and chemical characteristics of individual phenolic constituents may contribute to the antioxidant responses of these extracts [11,30,31]. In particular, differences in the phenolic profiles of *Rubus* spp. and *Vaccinium* spp., as identified by HPLC-PDA in the present study, may partly explain the contrasting responses observed in the FRAP and DPPH assays. In addition to phenolic constituents, other endogenous or non-phenolic antioxidants may also contribute to the overall antioxidant capacity of plant extracts [32,33]. However, these compounds were not determined in the present study, and therefore their contribution cannot be directly evaluated.

To further explore the potential relationship between the sampling-site characteristics and the measured bioactive properties, exploratory Spearman rank correlation analyses were performed using accession-level data. No significant correlations were observed between slope or altitude and TPC, FRAP, or DPPH radical-scavenging activity. A positive association was observed between altitude and TFC when all nine accessions were considered together (ρ = 0.683, *p* = 0.042). However, this association was not statistically significant when *Rubus* spp. and *Vaccinium* spp. were evaluated separately, with ρ = 0.600 (*p* = 0.285) and ρ = 0.200 (*p* = 0.800), respectively. These findings suggest that the overall altitude–TFC association may be influenced by differences between the two genera rather than representing an independent effect of altitude. Therefore, the present data do not provide sufficient evidence for an independent effect of altitude or slope on the measured bioactive and antioxidant properties.

Exploratory analysis of the topographic characteristics showed no statistically significant correlations between altitude or slope and TPC, TFC, FRAP, or DPPH radical-scavenging activity within either *Rubus* spp. or *Vaccinium* spp.; altitude showed a moderate positive association with TFC (ρ = 0.600, *p* = 0.285) and a strong negative association with DPPH SC_50_ (ρ = −0.821, *p* = 0.089), although neither relationship reached statistical significance. In *Vaccinium* spp., none of the correlations between altitude or slope and the measured parameters were statistically significant (*p* ≥ 0.200). When all nine accessions were considered together, altitude showed a nominal positive association with TFC (ρ = 0.683, *p* = 0.042), whereas no significant associations were observed for the other parameters. However, this overall association was not observed within either genus. The exploratory topographic analysis did not provide consistent evidence for an independent effect of altitude or slope on the measured bioactive and antioxidant properties. Although a nominal positive association between altitude and TFC was observed when all accessions were considered together, this relationship was not significant within either *Rubus* spp. or *Vaccinium* spp. This finding suggests that the overall association may be influenced by differences between the two genera rather than by altitude itself. Given the limited number of accession-level observations, these correlations should be interpreted cautiously.

The phenolic compositions of blueberry and blackberry extracts were determined using HPLC-PDA. Quantitative results of the analyses, which were based on 25 previously validated phenolic compounds, are presented in Table 3. The amounts of phenolic compounds were calculated in µg/100 g, and the results were presented under two main classes: phenolic acids and flavonoids.

The flavonoid compounds chrysin and pinocembrin were detected in varying concentrations in both wild samples. In contrast, apigenin was not detected in any sample. The literature reports that the major phenolic acid in Vaccinium species is chlorogenic acid, and that these species are also rich in anthocyanins. However, since anthocyanin compounds were not among the phenolic standards used in this study, these compounds were not analyzed [34,35,36,37]. Consistent with our study, the literature reports that ellagic acid is the major phenolic component in blackberry species and that these species have a rich composition of various anthocyanins responsible for their dark color [3,5].

Phenolic acids predominantly occur as hydroxybenzoic and hydroxycinnamic acid derivatives, each displaying characteristic distribution patterns across botanical matrices. Notably, *Rubus* spp. and *Vaccinium* spp. exhibited mutually exclusive phenolic acid fingerprints: ellagic acid and gallic acid predominated in blackberry extracts but were not detected in blueberry samples. In contrast, chlorogenic acid emerged as the dominant hydroxycinnamate in blueberry samples, whereas it was undetectable in the blackberry matrix.

Distinct phenolic acid distribution patterns were observed between the two berry groups. Chlorogenic acid was the predominant hydroxycinnamic acid in *Vaccinium* spp. [27], whereas it was not detected in *Rubus* spp. Conversely, gallic acid was detected at substantial levels in Rubus spp. but was not detected in Vaccinium samples under the applied analytical conditions [29]. Both compounds are well recognized for their antioxidant properties, with the high radical-scavenging capacity of gallic acid being largely associated with its three hydroxyl groups [29,38]. In addition, highly hydroxylated flavonoids, particularly anthocyanins such as cyanidin-3-*O*-glucoside [27] and flavan-3-ols such as catechin and epicatechin [29], may contribute substantially to the antioxidant capacity of berries. The presence of these phenolic compounds in the present samples may therefore contribute to their observed DPPH and FRAP activities.

Interestingly, in a recent study conducted on another wild berry species, white myrtle berry (*Myrtus communis* L.), chlorogenic acid was identified as the predominant phenolic acid, closely followed by gallic acid [39]. Although a similar analytical methodology was employed in that investigation, the fact that gallic acid was not detected under the applied analytical conditions in our high-altitude *Vaccinium* spp. samples highlights differences in phenolic acid profiles among wild berry taxa with overlapping secondary metabolite pathways. Our findings regarding the phenolic composition of *Rubus* spp. are in strong agreement with the literature. In a comprehensive study [40], 23 wild blackberry samples were analyzed, and a wide range of phenolic compounds, including anthocyanins, flavonols, hydroxycinnamic acids, and ellagic acid derivatives, was identified. Their findings further support the importance of cyanidin-3-*O*-glucoside and ellagic acid derivatives in the phenolic profile of Rubus fruits. This profile supports the importance of ellagic acid and its hydrolysable derivatives as characteristic phytochemicals of Rubus fruits.

## 3. Materials and Methods

### 3.1. Chemicals and Materials

The solvents of methanol, acetonitrile, diethyl ether, glacial acetic acid, and ethyl acetate used in the study were all of analytical purity and obtained from Merck, Darmstadt, Germany. All phenolic standards of gallic acid, protocatechuic acid, *p*-OH benzoic acid, chlorogenic acid, ellagic acid, *t*-cinnamic acid, ferulic acid, *p*-coumaric acid, galangin, quercetin, catechin hydrate, epicatechin, chrysin, apigenin, pinocembrin, hesperetin, caffeic acid, syringic acid, vanillic acid, rutin, rhamnetin, myricetin, catechin, luteolin, caffeic acid phenyl ester (CAPE) were supplied from Sigma-Aldrich Chemie (Taufkirchen, Germany). The chemicals used in the study. TPTZ, FeSO_4_∙7H_2_O, Folin–Ciocalteu’s reagent, and Quercetin were obtained from Merck.

### 3.2. Samples and Extractions

**Figure 1:**
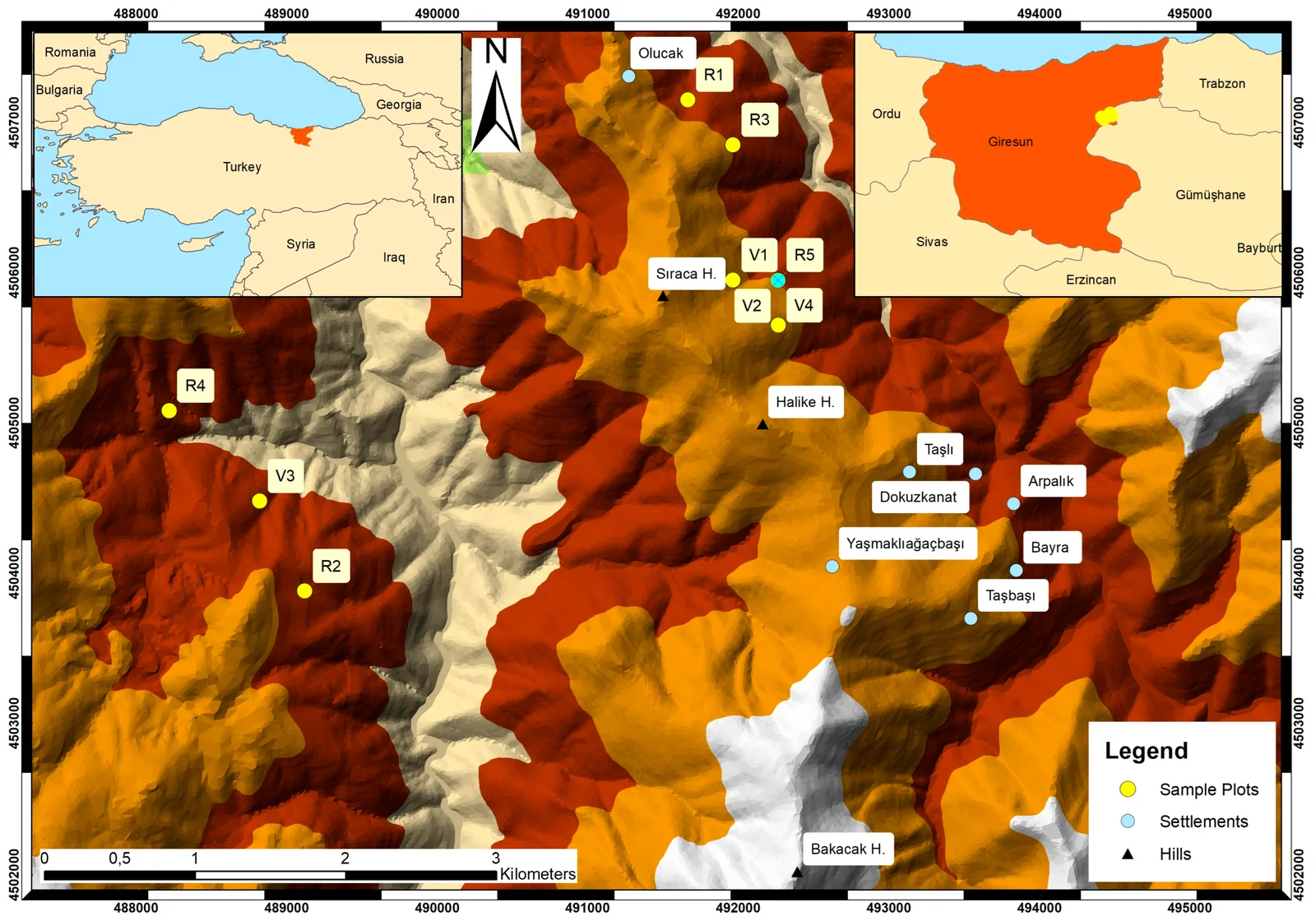
The map of the sampling region for *Rubus* spp. and *Vaccinium* spp.

In the present study, two wild berry taxa, Blackberry (*Rubus* spp.) and Blueberry (*Vaccinium* spp.), were evaluated. A total of nine wild berry accessions, comprising five blackberry and four blueberry accessions, were collected at full maturity in October 2023 from the Akılbaba Region (Giresun), located in the Eastern Black Sea region of Türkiye. The number of accessions reflected the naturally available and suitable wild berry material encountered at the sampling site. Botanical identification of the collected specimens was authenticated by Prof. Dr. Salih Terzioğlu from the Department of Forest Botany, Faculty of Forestry, Karadeniz Technical University. The blueberry specimens were identified as *Vaccinium arctostaphylos* L.; however, they are reported as *Vaccinium* spp. throughout the manuscript to maintain consistency with the taxonomic designation of the *Rubus* specimens, for which species-level identification could not be reliably resolved. The selected characteristics of the areas where the samples were obtained are provided in detail in Table 1. The map of the sampling region for *Rubus* spp. and *Vaccinium* spp. is presented in Figure 1. For each sampling site, a 400 m^2^ sampling area was selected based on the forest canopy closure conditions, as described in Table 1. The sampling strategy was based on predefined sampling areas rather than on a predetermined number of individual plants, as the target wild berry plants were naturally distributed within these areas and were not uniformly present throughout the entire sampling area. Fruits were collected from the berry-bearing plants encountered within the relevant parts of each defined sampling area. The collected fruits representing each accession were combined to obtain one composite sample for subsequent analysis. Thus, nine accession-level samples were obtained in total, comprising five *Rubus* (R1–R5) and four *Vaccinium* (V1–V4) accessions. An extract was prepared separately from each accession-level composite sample, resulting in nine individual extracts. Approximately 3 g of fresh material was thoroughly crushed in a mortar and extracted with 30 mL of 70% methanol. The mixture was shaken for 24 h at 25 °C. After 24 h, the extract was first filtered through ordinary filter paper and then further filtered using blue band filter paper. The filtrate was divided into two portions: the first portion was used for the antioxidant assays, while the second portion was used for HPLC analysis. At this stage, the samples were ready for antioxidant analysis and were stored at +4 °C until the analyses were performed [41].

### 3.3. Preparing the Extracts for HPLC Analysis

Phenolic composition was determined by HPLC–PDA following liquid–liquid extraction. Solvents were evaporated at 50 °C, and the residue was reconstituted in pH 2 water and extracted with diethyl ether and ethyl acetate. After solvent removal, samples were dissolved in methanol, filtered (0.45 µm) and analyzed by RP-HPLC–PDA using a C18 column and a gradient mobile phase of acetic acid in water and acetonitrile–water. Detection was performed at 250–360 nm, and phenolics were quantified using external calibration with standards, with results expressed as µg/100 g [42]. Phenolic composition was determined by HPLC-PDA following liquid–liquid extraction. Solvents were evaporated at 50 °C, and the residue was reconstituted in pH 2 water and extracted with diethyl ether and ethyl acetate. After solvent removal, the samples were dissolved in methanol and filtered through a 0.45 µm membrane filter. Chromatographic analysis was performed using a Shimadzu LC-20AT HPLC system (Shimadzu Corporation, Kyoto, Japan) equipped with a photodiode array detector (PDA) and an InertSustain C18 column (250 × 4.6 mm, 5 μm; GL Sciences, Tokyo, Japan). The mobile phase consisted of solvent A (70% acetonitrile in ultrapure water) and solvent B (2% acetic acid in ultrapure water). Gradient elution was performed over 50 min as follows: 0 min, 82% B; 5 min, 81% B; 10 min, 73% B; 14 min, 62% B; 25 min, 35% B; 40 min, 10% B; 40.01 min, 82% B; and 50 min, 82% B. The flow rate was 1.0 mL/min, the column temperature was maintained at 30 °C, the injection volume was 20 μL, and the autosampler temperature was maintained at 20 °C. The analytical procedure was based on the previously developed and validated method reported by Kara and Birinci 2024 [42], with the modifications described in the present study. The validation performance of the analytical method, including linearity (R^2^), limits of detection (LOD) and quantification (LOQ), relative error, and recovery for the individual phenolic standards, was established in the validation study reported by Kara and Birinci 2024 [42]. Identification of the targeted phenolic compounds was based on comparison of their retention times and UV spectra with those of the corresponding authentic standards. PDA detection was performed over the wavelength range of 250–360 nm, and the individual phenolic compounds were monitored at their respective analytical wavelengths (250, 280, 320, or 360 nm) according to the previously validated method [42]. Quantification was performed using external calibration curves prepared from the corresponding authentic standards, and the results were expressed as μg/100 g. The targeted HPLC-PDA analysis included 25 predefined phenolic standards. Of these, 15 compounds were detected in at least one sample, whereas 10 compounds were not detected in any of the samples under the applied analytical conditions. For each accession, the extract was subjected to a single chromatographic injection, and therefore no technical replicate injections were performed. The quantitative results for the 15 detected compounds are presented in Table 3.

### 3.4. Total Phenolic Content (TPC)

The total phenolic content (TPC) of the extracts was determined using the Folin–Ciocalteu assay [43]. Initially, 680 μL of the extract was combined with 400 μL of 0.2 N Folin–Ciocalteu’s reagent (Sigma Aldrich, Saint Louis, MO, USA. F9252). After a 3 -min incubation period, 400 μL of 10% sodium carbonate was added and the mixture was incubated for 2 h at room temperature. After the incubation, the absorbance was measured using a spectrophotometer (Thermo Scientific (Waltham, MA, USA) Evolution TM 201, UV-VIS Spectrophotometer, USA) at a wavelength of 760 nm. A gallic acid calibration curve (0.03125–1.00 mg/mL) was constructed, yielding the linear equation y = 1.5409x + 0.0668 (R^2^ = 0.9976).

### 3.5. Determination of Iron (III) Reducing Antioxidant Power (FRAP)

The Ferric Reducing Antioxidant Power (FRAP) assay was employed to assess the total antioxidant capacity of the samples [44]. The FRAP reagent was freshly prepared by combining 300 mM pH 3.6 sodium acetate buffer, 10 mM TPTZ. and 20 mM FeCl_3_ solutions in a 10:1:1 ratio. Subsequently, 1.5 mL of the FRAP reagent and 0.05 mL of the methanolic extract were added to a test tube. The mixture was incubated at 37 °C for 4 min; after that, the absorbance was measured at 595 nm. A standard curve was constructed using varying concentrations of FeSO_4_∙7H_2_O (31.25 µM–1000 µM), yielding the linear equation y = 0.0006x − 0.0123 (R^2^ = 0.9993). The antioxidant capacity of the samples was expressed as μmol FeSO_4_∙7H_2_O equivalent/g.

### 3.6. Determination of DPPH Radical Scavenging Activity

DPPH (2.2-diphenyl-1-picrylhydrazyl) free radical scavenging activity was determined in the methanolic extract [45]. Briefly, 750 μL of a 100 μM DPPH solution was mixed with an equal volume of the extract. The mixture was incubated for 45 min at room temperature. The absorbance was measured at 517 nm. The radical scavenging activity was expressed as SC_50_, the concentration of the sample required to reduce DPPH• radical concentration by 50%, with lower SC_50_ values indicating higher radical scavenging capacity. DPPH radical-scavenging activity was determined according to Molyneux, 2004 [46]. Different concentrations of the methanolic extracts were reacted with DPPH solution and incubated in the dark at room temperature for 50 min. Prior to the assay, preliminary dilution tests were performed for each accession to determine an appropriate extract concentration range. Accordingly, an accession-specific concentration range was used for each sample, and six concentration levels, including the corresponding blank/zero concentration, were prepared for each concentration–response curve. Absorbance was measured at 517 nm. For each accession, a separate concentration–response graph was generated, and the SC_50_ value was calculated from the corresponding regression equation as the extract concentration required to achieve 50% DPPH radical-scavenging activity. Each concentration was analyzed in triplicate. Results were expressed as SC_50_ values (mg/mL), with lower SC_50_ values indicating stronger radical-scavenging activity.

### 3.7. Total Flavonoid Content (TFC)

The total flavonoid content (TFC) of the methanolic extract was determined using the aluminum nitrate colorimetric method. Briefly, 250 μL of the extract was mixed with 50 μL of 10% Al (NO_3_)_3_ and 50 μL of 1 M ammonium acetate (NH_4_CH_3_COO). The reaction mixture was brought to a final volume of 3 mL with 99% ethanol and incubated at room temperature for 40 min. Absorbance was then measured at 415 nm against a blank solution. Quercetin solutions (0.0078–0.5 mg QE/mL) were used to construct the calibration curve (the calibration equation was y = 6.5932∙x + 0.0604, R^2^ = 0.9923), and TFC was expressed as mg quercetin equivalents (QE) per g of sample [47]. 

### 3.8. Statistics

All analyses were performed in triplicate using the same extract from each accession. For HPLC-PDA analysis, each accession was analyzed using a single chromatographic injection; therefore, no technical replicate injections were performed for the phenolic compound determination. These triplicate measurements represented parallel analytical measurements of the same extract and were not considered independent biological observations. Accordingly, each accession was considered the experimental unit, resulting in five *Rubus* spp. and four *Vaccinium* spp. accession-level experimental units. Results are expressed as mean ± standard deviation based on accession-level means. Statistical analyses were performed using IBM SPSS Statistics (version 26; IBM Corp., Armonk, NY, USA). Differences between *Rubus* spp. and *Vaccinium* spp. in total phenolic content (TPC), total flavonoid content (TFC), ferric reducing antioxidant power (FRAP), and DPPH radical-scavenging activity were assessed using the two-sided nonparametric Mann–Whitney U test based on accession-level means. To explore potential associations between the topographic characteristics of the sampling sites and the measured bioactive and antioxidant parameters, Spearman rank correlation analyses were additionally performed using accession-level means for altitude and slope. These correlation analyses were considered exploratory, and the relationships were evaluated both across all accessions and within each genus. Exact *p*-values are reported, and *p* < 0.05 was considered statistically significant.

Spearman rank correlation analyses were additionally performed to explore potential associations between altitude and slope and the measured TPC, TFC, FRAP, and DPPH parameters. The analyses were performed using accession-level means and evaluated both across all accessions and separately within *Rubus* spp. and *Vaccinium* spp.

## 4. Conclusions

*Rubus* spp. and *Vaccinium* spp. represent two of the most ecologically widespread and economically prominent wild berry genera, renowned for their distinct sensory profiles and substantial bioactive potential. In the present study, the individual phenolic compositions and antioxidant capacities of wild blackberry (*Rubus* spp.) and blueberry (*Vaccinium* spp.) populations harvested from the high-altitude plateau habitats of the Eastern Black Sea region (Türkiye) were comparatively characterized. The chromatographic and spectrophotometric findings demonstrated notable differences in the phenolic profiles of the sampled accessions, particularly in their individual phenolic acid and flavonoid distributions. Interestingly, despite marked divergences in their individual phenolic profiles, the antioxidant responses were assay-dependent, with comparable FRAP values but significantly stronger DPPH radical-scavenging activity in *Rubus* spp. Blackberries were particularly rich in ellagic and gallic acids, whereas blueberries were characterized by a higher abundance of chlorogenic acid. These findings highlight the potential of wild high-altitude berry germplasms as sources of selected natural bioactive compounds and antioxidants, providing a basis for further studies on food-compatible extraction, bioaccessibility, and their potential nutraceutical applications. However, further studies addressing bioavailability, biological efficacy, safety, and in vivo effects are required before such applications can be proposed.

## Figures and Tables

**Table 1:** Selected characteristics of *Vaccinium* spp. and *Rubus* spp. samples and their collection regions.

Sample	Code	X Coordinate	Y Coordinate	Slope (%)	Altitude (m)
Blackberry (*Rubus* spp.)	R1	491666	4507149	45	1717
	R2	489120	4503879	30	1695
	R3	491966	4506849	43	1741
	R4	488220	4505079	60	1588
	R5	492266	4505949	34	1768
Blueberry (*Vaccinium* spp.)	V1	491966	4505949	38	1810
	V2	492264	4505947	34	1769
	V3	488820	4504479	42	1609
	V4	492266	4505649	35	1831

**Table 2:** Phenolic content and antioxidant properties of the *Rubus* spp. and *Vaccinium* spp.

		TPC (mg GAE/g FW)	TFC (mg QE/g FW)	FRAP (µmolFeSO_4_∙7H_2_O E/g FW)	DPPH (SC_50_ mg/mL)
Blackberry (*Rubus* spp.)	R1	4.79 ± 0.22	0.11 ± 0.00	97.38 ± 0.73	0.48 ± 0.02
	R2	4.25 ± 0.56	0.02 ± 0.00	83.91 ± 0.35	0.50 ± 0.01
	R3	4.23 ± 0.43	0.06 ± 0.04	88.93 ± 1.06	0.50 ± 0.01
	R4	4.80 ± 0.21	0.04 ± 0.01	101.25 ± 2.24	0.54 ± 0.02
	R5	4.36 ± 0.76	0.07 ± 0.01	108.95 ± 1.85	0.42 ± 0.04
	**Mean**	**4.49 ± 0.29**	**0.06 ± 0.03**	**96.08 ± 9.92**	**0.49 ± 0.04**
Blueberry (Vaccinium spp.)	V1	5.57 ± 0.10	0.57 ± 0.01	77.98 ± 2.62	2.58 ± 0.06
	V2	4.35 ± 0.52	0.58 ± 0.02	110.20 ± 0.96	2.74 ± 0.04
	V3	7.35 ± 0.67	0.34 ± 0.01	95.30 ± 1.75	2.61 ± 0.15
	V4	6.16 ± 0.20	0.38 ± 0.01	97.70 ± 2.15	2.77 ± 0.21
	**Mean**	**5.86 ± 1.25**	**0.47 ± 0.13**	**95.30 ± 13.26**	**2.68 ± 0.09**
*p*-value		0.1111	0.0159	1.0000	0.0159

**Table 3:** Phenolic composition of the *Rubus* spp. and *Vaccinium* spp.

		R1	R2	R3	R4	R5	V1	V2	V3	V4
***Phenolic acids (µg/100 g)***	**Hydroxybenzoic acids**									
	*p*-OH Benzoic acid	295.1	n.d.	n.d.	n.d.	207.3	n.d.	n.d.	n.d.	n.d.
	Protocatechuic acid	313.0	219.0	121.8	205.9	291.8	n.d.	n.d.	n.d.	n.d.
	Gallic acid	1005.3	738.9	432.1	357.0	1132.6	n.d.	n.d.	n.d.	n.d.
	**Hydroxycinnamic acids**									
	Chlorogenic acid	n.d.	n.d.	n.d.	n.d.	n.d.	127975.2	122900.8	72393	91160.6
	Ellagic acid	1415.7	2046.0	5306.8	3371.4	312.7	n.d.	n.d.	n.d.	n.d.
	*t*-cinnamic acid	82.2	3277.2	n.d.	n.d.	60.3	n.d.	n.d.	n.d.	n.d.
	Ferulic acid	n.d.	n.d.	n.d.	n.d.	n.d.	161.9	1010.0	557.2	n.d.
	*p*-Coumaric acid	n.d.	n.d.	n.d.	n.d.	1.710				
***Flavonoids (µg/100 g)***	**Flavonol**									
	Galangin	n.d.	4601.8	107.1	n.d.	n.d.	508.3	281.7	245.3	313.2
	Quercetin	183.1	n.d.	n.d.	n.d.	177	n.d.	n.d.	n.d.	n.d.
	**Flavan-3-ols**									
	Catechin hydrate	n.d.	n.d.	n.d.	290.9	646.1	n.d.	n.d.	n.d.	n.d.
	Epicatechin	n.d.	n.d.	n.d.	n.d.	158.4	n.d.	n.d.	n.d.	n.d.
	**Flavones**									
	Chrysin	139.5	4468.2	95.8	97.8	761.2	407.3	342.3	322.7	364.2
	**Flavanones**									
	Pinocembrin	309.1	4522.9	98.4	94.4	576.1	323.8	370.4	255.8	332.2
	Hesperetin	175.7	3576	n.d.	37.2	125.6	n.d.	n.d.	n.d.	n.d.

Note: HPLC-PDA values represent the concentrations determined from one extract prepared separately from each accession and subjected to a single chromatographic determination. Therefore, replicate-based standard deviations are not reported for the chromatographic measurements. n.d.: Apigenin, caffeic acid, syringic acid, vanillic acid, rutin, myricetin, catechin, luteolin, caffeic acid phenyl ester (CAPE), and rhamnetin were not detected in any of the samples under the applied analytical conditions.

## Data Availability

The data supporting the findings of this study are available from the corresponding author upon reasonable request.

## Abbreviations

FRAP	Ferric Reducing Antioxidant Power
TPTZ	2,4,6-tripyridyl-s-triazine
DPPH	2,2-Diphenyl-1-picrylhydrazyl
SC_50_	Radical Scavenging Concentrations
CAPE	Caffeic Acid Phenethyl Ester
